# Effect of HHH-Therapy on Regional CBF after Severe Subarachnoid Hemorrhage Studied by Bedside Xenon-Enhanced CT

**DOI:** 10.1007/s12028-017-0439-y

**Published:** 2017-10-05

**Authors:** Henrik Engquist, Elham Rostami, Elisabeth Ronne-Engström, Pelle Nilsson, Anders Lewén, Per Enblad

**Affiliations:** 1 Dept of Neuroscience/Neurosurgery, Uppsala University, Uppsala, Sweden; 2 Dept of Surgical Sciences/Anesthesia and Intensive Care, Uppsala University Hospital, 751 85 Uppsala, Sweden

**Keywords:** Subarachnoid hemorrhage (SAH), Delayed cerebral ischemia (DCI), Cerebral blood flow (CBF), HHH-therapy (Triple-H), Xenon CT (XeCT)

## Abstract

**Background:**

Management of delayed cerebral ischemia (DCI) following subarachnoid hemorrhage (SAH) is difficult and still carries controversies. In this study, the effect of therapeutic hypervolemia, hemodilution, and hypertension (HHH-therapy) on cerebral blood flow (CBF) was assessed by xenon-enhanced computerized tomography (XeCT) hypothesizing an increase in CBF in poorly perfused regions.

**Methods:**

Bedside XeCT measurements of regional CBF in mechanically ventilated SAH patients were routinely scheduled for day 0–3, 4–7, and 8–12. At clinical suspicion of DCI, patients received 5-day HHH-therapy. For inclusion, XeCT was required at 0–48 h before start of HHH (baseline) and during therapy. Data from corresponding time-windows were also collected for non-DCI patients.

**Results:**

Twenty patients who later developed DCI were included, and twenty-eight patients without DCI were identified for comparison. During HHH, there was a slight nonsignificant increase in systolic blood pressure (SBP) and a significant reduction in hematocrit. Median global cortical CBF for the DCI group increased from 29.5 (IQR 24.6–33.9) to 38.4 (IQR 27.0–41.2) ml/100 g/min (*P* = 0.001). There was a concomitant increase in regional CBF of the worst vascular territories, and the proportion of area with blood flow below 20 ml/100 g/min was significantly reduced. Non-DCI patients showed higher CBF at baseline, and no significant change over time.

**Conclusions:**

HHH-therapy appeared to increase global and regional CBF in DCI patients. The increase in SBP was small, while the decrease in hematocrit was more pronounced, which may suggest that intravascular volume status and rheological effects are of importance. XeCT may be potentially helpful in managing poor-grade SAH patients.

## Introduction

The complex mechanisms leading to secondary brain injury and the sometimes devastating course of delayed cerebral ischemia (DCI) [1] after aneurysmal subarachnoid hemorrhage (SAH) are still not well understood [2]. Improved techniques for early surgical or endovascular interventions and general improvement of the neurointensive care (NIC) have reduced mortality and morbidity in these patients during the last decades [3]. Still there is little evidence supporting prophylactic strategies to minimize the risk of DCI and also concerning therapeutic interventions when DCI is suspected [4]. In modern NIC, clinical surveillance combined with multimodal systemic and cerebral monitoring is used to aid the early detection of avoidable factors and also DCI [5]. There is, however, no consensus regarding the best tools to guide the therapy at suspicion of DCI [6].

After early repair of the aneurysm, most guidelines propose fluid therapy to ensure normovolemia or mild hypervolemia, monitoring of blood pressure and vasoactive support if necessary to avoid hypotension [7, 8]. At suspicion of DCI, therapeutic hypervolemia, hemodilution, and hypertension (HHH-therapy) [9] has been widely used aiming to increase cerebral blood flow (CBF) in ischemic regions. Controversy exists concerning the actual effects of the different components of this intervention [10]. In the standard NIC protocol for SAH patients at Uppsala University Hospital, therapeutic interventions on suspicion of DCI include cautious institution of HHH-therapy with moderate blood pressure targets and frequent reevaluation to avoid side effects and complications.

The aim of this study was to evaluate the effects of HHH-therapy on CBF, testing the hypothesis that HHH-therapy increases global CBF and also regional CBF (rCBF) in areas at risk of ischemia. Bedside xenon-enhanced computerized tomography (XeCT) was used to assess rCBF prior to clinical suspicion of DCI and during the treatment to resolve DCI.

## Materials and Methods

### Patients

Patients diagnosed with severe spontaneous SAH admitted to Uppsala University Hospital during the time period 2013–2016 were prospectively enrolled in the study. The diagnosis of SAH was verified by CT. As ventilator treatment is a prerequisite for bedside XeCT in our setting, only patients requiring intubation due to their neurological state at admission or neurological deterioration day 0–1 were included, i.e., patients with more severe SAH. Exclusion criteria were severe intracranial hypertension, deep sedation with thiopental, respiratory problems requiring FiO_2_ >0.6, the absence of informed consent, and futility or “do not resuscitate” order.

### CBF Measurements: protocol

According to our standard NIC protocol, CBF measurements were scheduled for three time intervals: day 0–3, 4–7, and 8–12. Patients with clinical diagnose of DCI received HHH-therapy as described below. For inclusion in this study, a XeCT measurement within 0–48 h before the start of HHH-therapy was required (used as baseline), and the intention was to perform the next XeCT measurement during ongoing therapy when logistically possible. Data were also collected for non-DCI patients with corresponding XeCT measurements in the same time-windows as a reference group for comparison.

### Xenon CT CBF Measurements: Method

**Fig. 1:**
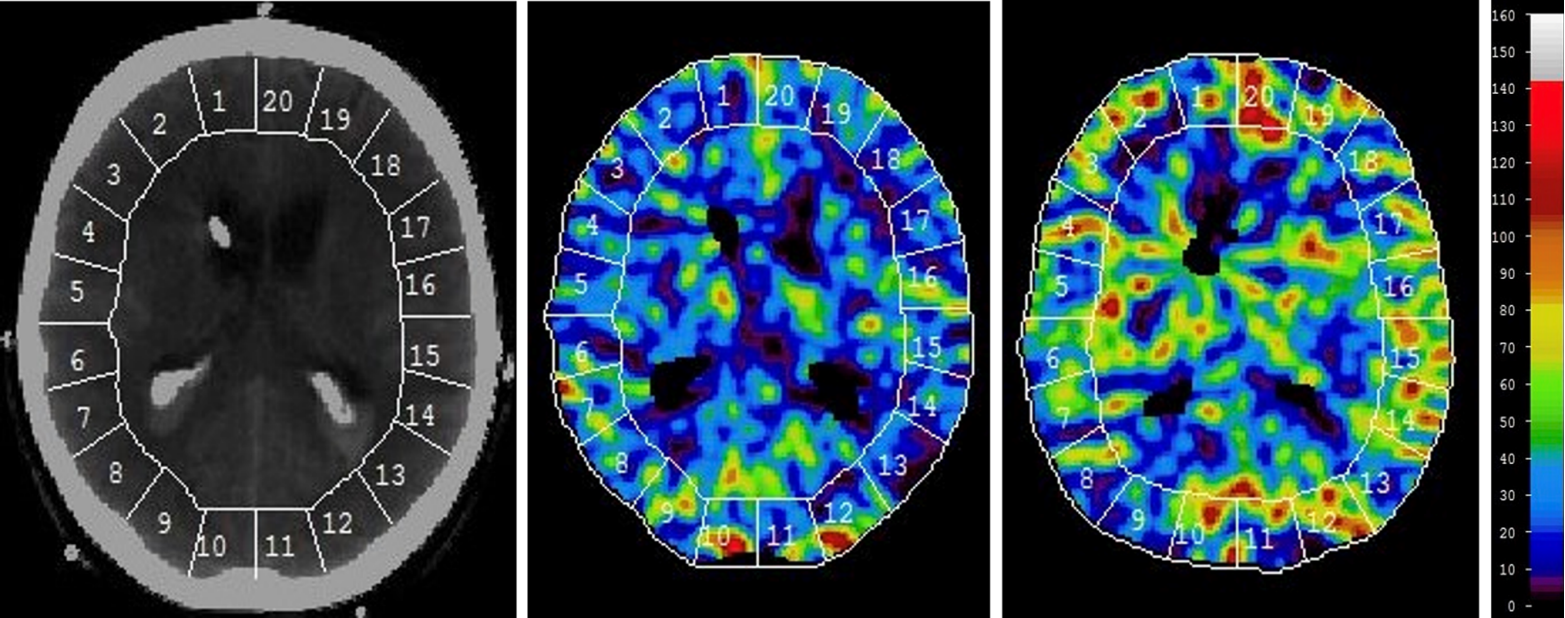
Example of XeCT measurements before and during HHH-therapy (one of three scan levels). Global and regional cortical CBF (ml/100 g/min) was calculated as mean of CBF in the corresponding regions of interest (ROIs) at three scan levels of the brain for each patient. Global CBF—ROI 1–20. Right anterior cerebral artery territory—ROI 1–2, right middle cerebral artery territory—ROI 3–8, right posterior cerebral artery territory—ROI 9–10 and equally for ROI 11–20 on the *left side*. *CBF* cerebral blood flow, *HHH-therapy* therapeutic hypervolemia, hemodilution and hypertension, *XeCT* xenon-enhanced computerized tomography

The XeCT procedures in our NIC unit are conducted as described by Gur et al. and Yonas et al. [11–14], using inhaled xenon as an inert contrast agent during the CT scans. Since xenon is radiopaque, lipid soluble, and crosses the blood brain barrier, its concentration in the cerebral tissue can be measured by CT. Blood flow calculation is based on Kety’s [15, 16] application of the Fick principle; blood flow is proportional to inert gas uptake in tissue. Mobile equipment allows bedside measurements in the NIC unit, which facilitates the clinical use of XeCT [17]. Stable (non-radioactive) xenon at a concentration of 28% is administered to the patients breathing circuit for 4.5 min using the Enhancer 3000 and computer software (Diversified Diagnostic Products Inc, Huston, USA). CT scans during the xenon inhalation are obtained by the CereTom (Neurologica, Boston, USA). The xenon delivery and CT scans are synchronized by the computer software, and the resulting radiologic tissue enhancement of the xenon wash-in enables CBF to be calculated (ml/100 g/min) and plotted as color-coded maps in scans at four different levels of the brain (eight scans per level, two at baseline and six enhanced during xenon wash-in). Levels with extensive artifacts from bone structures or coils/clips were excluded from calculation. Typically, three scan levels could be used for further calculations in each patient. Mean blood flow in each of twenty evenly distributed cortical regions of interest (ROIs) was calculated (Fig. 1) for every level [13], resulting in a total of sixty ROIs. Typical area of each ROI was 350–450 mm^2^. ROIs in areas of hematoma or with artifacts from ventricular drains were excluded.

### Neurointensive Care

Management of all patients followed our standard NIC procedures for SAH as earlier described by Ryttlefors et al. [5], where the cornerstones are continuous multimodal monitoring of physiological and biochemical parameters and cautious clinical observation for avoidable factors with prompt interventions to minimize secondary brain injury. Sedation with propofol (Propolipid^®^, Fresenius Kabi AB, Uppsala, Sweden) is titrated in the interval 0–4 mg/kg/h, and morphine (Morfin, Meda AB, Solna, Sweden) is administered as needed. Patients with suspicion of hydrocephalus or altered level of consciousness receive a ventriculostomy catheter for monitoring of intracranial pressure (ICP) and drainage of cerebrospinal fluid. Elevated ICP (>20 mmHg) is treated with open ventricular drainage against a pressure level of 15 mmHg. The general policy is to treat aneurysms early with endovascular coil embolization as first choice when possible or surgical clipping. After the aneurysm repair, patients are kept mildly hypervolemic if not contraindicated by intracranial mass effect or elevated ICP. The volume status is maintained by fluid administration in the higher normal range with addition of albumin infusion (Flexbumin^®^ 200 mg/ml, Baxter AG, Vienna, Austria) if needed and monitored by central venous pressure, clinical evaluation and rigorous fluid balance calculation. Nimodipine (Nimotop^®^, Bayer Pharma AG, Berlin, Germany) is given from day one and onwards.

### Detection of DCI and HHH-Therapy

To detect DCI, patients were monitored for general and focal neurological changes by repeated wake-up tests [18]. In the case of neurological deterioration, CT and laboratory tests were performed to rule out intracranial mass, manifest infarction, hydrocephalus, and meningitis. If DCI was concluded in the absence of other causes of deterioration, HHH-therapy to augment CBF was initiated for 5 days if not contraindicated by the intracranial situation, congestive heart failure, fluid overload or severe respiratory problems. Patients received daily infusions of 500 ml dextran solution (Rheomacrodex^®^, Meda AB, Solna, Sweden) and 200 ml albumin (Flexbumin^®^ 200 mg/ml, Baxter AG, Vienna, Austria) and were kept in supine position. In our standard NIC protocol, emphasis is on avoiding severe side effects and the blood pressure targets during HHH-therapy are moderate. The systolic blood pressure (SBP) was closely monitored and kept above 140 mmHg using vasoactive agents if needed. As first line, dobutamine (Hospira, Lake Forest, IL, USA) was used for inotropy or, if insufficient, norepinephrine (Noradrenalin, Hospira Nordic AB, Stockholm, Sweden) was added as vasopressor. The clinical effect of the HHH-therapy was repeatedly evaluated.

### XeCT Parameters

Global cortical CBF (ml/100 g/min) was calculated as mean of all ROIs at all scan levels used (Fig. 1). Regional CBF for each vascular territory was calculated as mean of the corresponding ROIs at all scan levels used (Fig. 1): right anterior cerebral artery—ROI 1–2, right middle cerebral artery—ROI 3–8, right posterior cerebral artery—ROI 9–10 and equally for ROI 11–20 on the left side. The vascular territory with lowest CBF was identified as the worst vascular territory in each patient.

To detect and quantify the extent of areas with low blood flow and near-ischemic flow, thresholds for local CBF were set to 20 and 10 ml/100 g/min [19, 20]. The percentage of cortical ROI area with local CBF below these thresholds was then calculated as sum of ROI area (mm^2^) with local CBF below the threshold divided by the total analyzed ROI area in each patient.

### Short-Term Outcome Parameters

Clinical course outcome was defined as neurological state at discharge from the NIC unit and determined as good (responding to commands, GCS motor 6), poor (unconscious, GCS motor ≤5), or *dead.*

Cerebral infarcts visible on follow-up CT at 12 days or later after admission to the NIC unit were categorized as <20 mm, 20–39 mm, >40 mm, or multiple.

### Statistical Methods

SPSS statistics 23.0 software (IBM Corp, Armonk, NY, USA) was used for statistical analyses of the collected data. Differences in systemic physiological parameters between measurements were tested by paired samples *t* test. CBF data for groups of patients are presented as median values and interquartile range because of non-normal distribution. Differences in CBF parameters between related samples were tested by Wilcoxon signed-ranks test, and between independent samples by Mann–Whitney *U* test. Statistical significance level was set at *P* < 0.05.

## Results

**Fig. 2:**
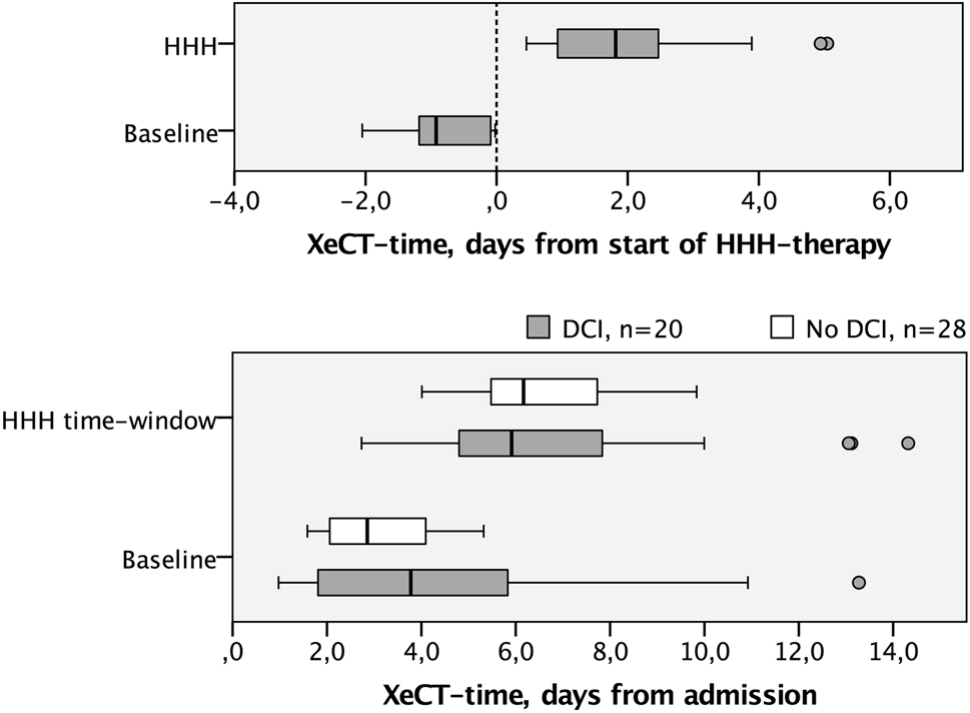
Time for XeCT measurements in relation to the start of HHH-therapy for DCI patients (*above*). Time for XeCT at baseline and during HHH-therapy in relation to admission to neurosurgical intensive care for DCI patients (*gray*) and for non-DCI patients (*white*) at corresponding time-windows (*below*). *DCI* delayed cerebral ischemia, *HHH-therapy* therapeutic hypervolemia, hemodilution and hypertension, *XeCT* xenon-enhanced computerized tomography

A total of 119 intubated SAH patients underwent one or more XeCT measurements during the time period 2013–2016. During this period, 20 patients with clinical suspicion of DCI were identified where XeCT measurements of CBF had been taken within 0–48 h before the initiation of HHH-therapy (baseline), and during the 5-day HHH-therapy (Fig. 2). Twenty-eight SAH patients with no suspicion of DCI, who had corresponding XeCT measurements in the same time-windows, were identified as a group for comparison of the natural course (Fig. 2). The characteristics of these groups are presented in Table 1. The median time-point for initiation of HHH-therapy was 4.38 days (IQR 2.75–5.97) after the admission to the NIC unit.

**Table 1:** Characteristics of patients in the DCI group who received HHH-therapy and the non-DCI group

		DCI		No DCI	
Characteristic		n	(%)	n	(%)
Patients	n	20		28	
Gender	F	12	(60)	19	(68)
Age, years	Mean [range]	58	[40–75]	60	[28–84]
Hunt and Hess grade at admission/first XeCT^1^	I	0/0		2/0	(7/0)
	II	5/0	(25/0)	1/0	(4/0)
	III	5/1	(25/5)	7/8	(25/29)
	IV	7/18	(35/90)	14/18	(50/64)
	V	3/1	(15/5)	4/2	(14/7)
CT Fisher group	2	0		1	(4)
	3	6	(30)	5	(18)
	4	14	(70)	22	(79)
Treatment modality	Endovascular	18	(90)	18	(64)
	Surgical clip	1	(5)	9	(32)
	None	1	(5)	1	(4)

^1^ Neurological state according to Hunt and Hess assessed both at admission and at the time of the first XeCT. *DCI* delayed cerebral ischemia, *HHH-therapy* therapeutic hypervolemia, hemodilution and hypertension, *XeCT* xenon-enhanced computerized tomography

### Hemodynamics and Ventilation

Hemodynamic parameters and ventilation were clinically stable *during the XeCT procedures*. The alterations from start to end in all procedures (*N* = 96) were small; mean arterial pressure (MAP) mean 92.5 mmHg (CI 90.0–95.0) versus 89.9 (CI 87.6–92.2), ICP mean 14.3 mmHg (CI 13.2–15.4) versus 14.4 (CI 13.5–15.4), PCO_2_ mean 5.41 kPa (CI 5.29–5.53) versus 5.51 (CI 5.40–5.63). No adverse events were observed in relation to the procedures.

SBP, MAP, cerebral perfusion pressure (CPP), pCO_2_, and hematocrit at start of the XeCT measurements *at the different time-windows for the* DCI and non-DCI groups are presented in Table 2. The mean SBP was slightly elevated during HHH-therapy in the DCI group, from 151.2 mmHg (CI 142.1–160.3) to 157.3 mmHg (CI 150.7–163.8), but the difference did not reach statistical significance. Among the DCI patients, hematocrit was significantly reduced during HHH-therapy compared to baseline (Table 2).

**Table 2:** Systemic hemodynamic parameters, ventilation, sedation, and vasoactive medication at the time of XeCT measurements (upper part). Calculated XeCT CBF parameters (lower part)

	DCI (*n* = 20) Baseline		DCI During HHH		No DCI (*n* = 28) Baseline		No DCI Day 5–8	
	Mean	(CI)	Mean	(CI)	Mean	(CI)	Mean	(CI)
SBP mmHg	151.2	(142.1–160.3)	157.3	(150.7–163.8)	150.0	(143.5–156.4)	152.9	(145.9–159.8)
		*P* = 0.105						
MAP mmHg	95.4	(87.1–103.7)	94.0	(88.7–99.4)	90.3	(87.0–93.6)	92.5	(88.2–96.7)
CPP mmHg	80.9	(72.4–89.3)	79.7	(74.3–85.1)	78.6	(74.3–82.9)	79.0	(74.8–83.3)
Hematocrit %	36.4	(34.7–38.0)	31.7	(30.2–33.2)	33.9	(32.6–35.3)	32.5	(31.4–33.6)
		*P* < 0.001*						
pCO_2_ kPa	5.20	(4.96–5.44)	5.34	(5.10–5.57)	5.35	(5.14–5.57)	5.71	(5.45–5.97)
Sedation dose, propofol mg/kg/h	2.58	(2.15–3.02)	2.57	(2.16–2.98)	2.70	(2.13–3.26)	2.81	(2.22–3.40)

	n	[range]	n	[range]	n	[range]	n	[range]
Dobutamine, n [range ug/kg/min]	0	[–]	5	[1.1–12.0]	4	[1.6–4.0]	3	[1.0–6.2]
Norepinephrine, n [range ug/kg/min]	1	[0.06]	2	[0.05–0.15]	3	[0.01–0.12]	2	[0.05–0.08]

	Median	(IQR)	Median	(IQR)	Median	(IQR)	Median	(IQR)
glob CBF ml/100 g/min	29.5	(24.6–33.9)	38.4	(27.0–41.2)	34.9	(29.0–41.7)	36.5	(28.0–42.3)
		*P* = 0.001*						
% ROI area [rCBF < 20 ml/100 g/min]	26.2	(13.4–44.5)	8.55	(2.4–34.8)	11.9	(3.2–23.0)	9.1	(2.1–29.2)
		*P* = 0.019*						
% ROI area [rCBF < 10 ml/100 g/min]	6.7	(0.0–11.0)	0.0	(0.0–5.0)	0.7	(0.0–4.6)	0.7	(0.0–4.9)
		*P* = 0.056						
rCBF worst territory ml/100 g/min	19.6	(15.0–24.2)	27.3	(17.8–34.1)	27.2	(20.8–35.4)	25.8	(17.4–31.4)
		*P* = 0.006*						
Index [rCBFworst/best hemisph]	0.59	(0.46–0.75)	0.70	(0.55–0.83)	0.70	(0.60–0.79)	0.63	(0.52–0.73)
		*P* = 0.040*				*P* = 0.029*		

* Indicates *P* < 0.05. *CBF* cerebral blood flow, *CI* confidence interval, *CPP* cerebral perfusion pressure, *DCI* delayed cerebral ischemia, *HHH-therapy* therapeutic hypervolemia, hemodilution and hypertension, *IQR* interquartile range, *MAP* mean arterial pressure, *rCBF* regional cerebral blood flow, *ROI* region of interest, *SBP* systolic blood pressure

The use of inotropes and vasopressors was low in all groups. The number of patients and dose range for each group are also presented in Table 2.

### Global Cortical CBF

**Fig. 3:**
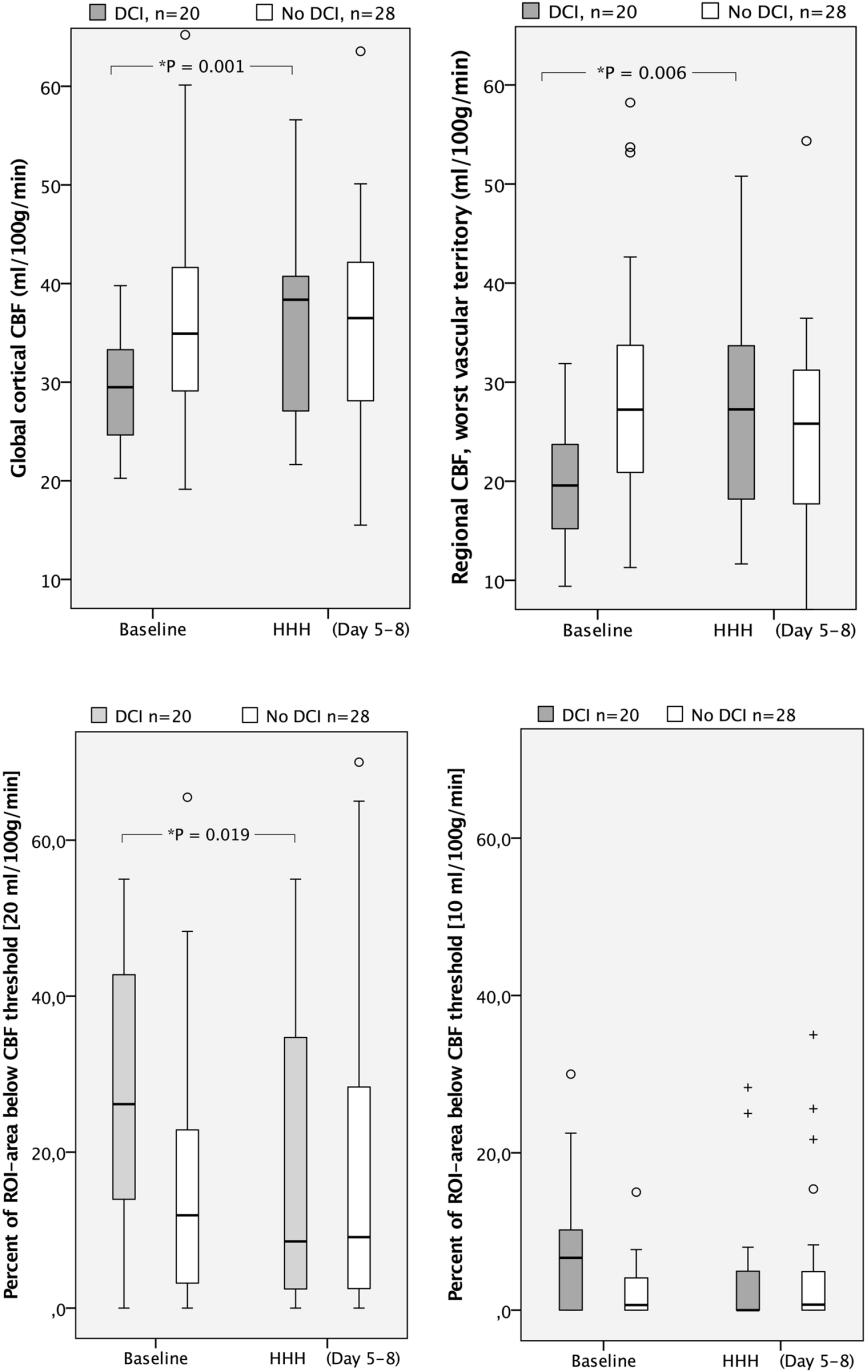
*Boxplots* of global cortical CBF and regional CBF of the worst vascular territory for patients clinically diagnosed with DCI at baseline and during HHH-therapy, and patients with no suspicion of DCI at corresponding time-windows (*above*). Proportion (%) of ROI area with local CBF below thresholds of 20 and 10 ml/100 g/min, respectively, for DCI and non-DCI patients (*below*). *CBF* cerebral blood flow, *DCI* delayed cerebral ischemia, *HHH-therapy* therapeutic hypervolemia, hemodilution and hypertension, *ROI area* region of interest area

Results of the XeCT measurements are presented in Table 2 and Fig. 3. Global cortical CBF at baseline was significantly lower in the DCI group compared to the non-DCI group; median 29.5 ml/100 g/min (IQR 24.6–33.9) versus 34.9 (IQR 29.0–41.7) (*P* = 0.005).

During HHH-therapy, the DCI group showed an increase in median global cortical CBF from 29.5 (IQR 24.6–33.9) to 38.4 (IQR 27.0–41.2) ml/100 g/min (*P* = 0.001), while no significant change over time was seen in the non-DCI group. No statistical analyses were performed for differences between DCI patients *during* HHH-therapy and non-DCI patients at the corresponding time-window.

### Regional CBF and CBF Heterogeneity

At baseline, there were also significant differences in regional CBF parameters between the DCI and non-DCI groups (Table 2; Fig. 3); median rCBF of the worst vascular territory for the DCI group was 19.6 (IQR 15.0–24.2) compared to 27.2 (20.8–35.4) ml/100 g/min for the non-DCI group (*P* = 0.005). Concerning regions with low or near-ischemic CBF, the DCI group had a larger proportion of ROI area with blood flow below 20 ml/100 g/min (median 26.2 vs 11.9%, *P* = 0.005) and flow below 10 ml/100 g/min (median 6.7 vs 0.7%, *P* = 0.023) compared to the non-DCI group.

During HHH-therapy, median rCBF of the worst vascular territory for the DCI group increased from 19.6 (IQR 15.0–24.2) to 27.3 (IQR 17.8–34.1) ml/100 g/min (*P* = 0.006) (Table 2; Fig. 3), and the proportion of ROI area with local blood flow below the threshold of 20 ml/100 g/min was reduced from median 26.2% at baseline to 8.55% (*P* = 0.019). The area with flow below 10 ml/100 g/min was small already at baseline but tended to decrease (from 6.7 to 0.0%, *P* = 0.056). The relative blood flow in the worst vascular territory compared to the best hemispheric blood flow (CBF index of worst vascular territory) showed a small but statistically significant increase during HHH-therapy. In the non-DCI group, there were no significant changes from baseline to the second time-window regarding rCBF in the worst vascular territory or the proportion of ROI area with local blood flow below the specified thresholds. In this group, there was a small, but statistically significant reduction in the CBF index of worst vascular territory between the two measurements.

### Short-Term Outcome; Clinical Course and Cerebral Infarcts

**Fig. 4:**
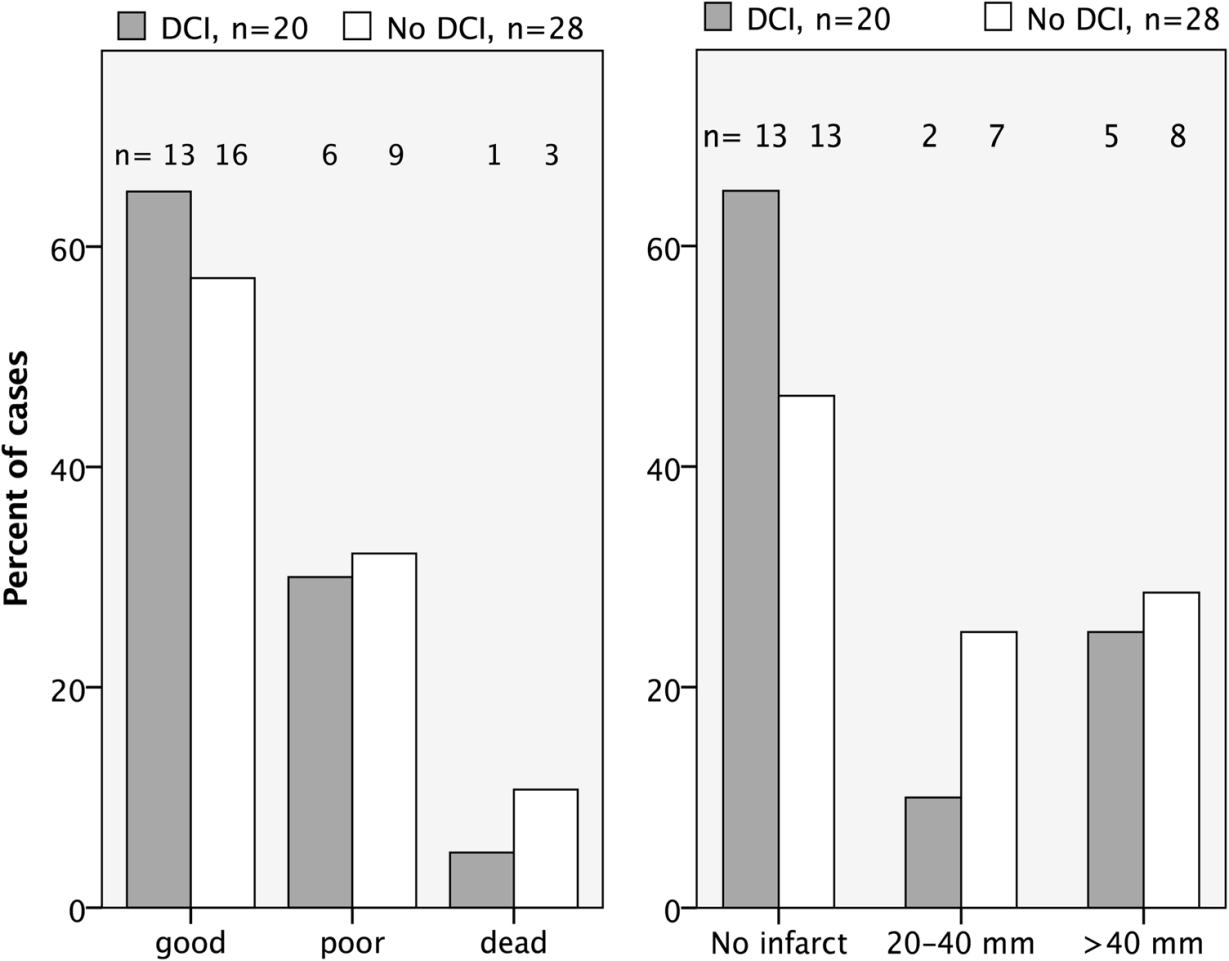
Clinical course outcome for patients in the DCI and non-DCI groups; neurological state at discharge from the NIC unit (*good*—responding to commands and GCS motor 6, *poor*—unconscious and GCS motor ≤5, *dead*). Infarcts visible on follow-up CT > day 12. *CT* computerized tomography, *DCI* delayed cerebral ischemia, *GCS motor* motor component of Glasgow coma scale, *NIC* neuro intensive care

Short-term outcome parameters are presented in Fig. 4. The proportion of patients with favorable clinical course outcome was 65% in the HHH-treated DCI group and 57% in the non-DCI group. The proportion of patients where no infarcts >20 mm were detected at follow-up CT was 65% in the DCI group and 46% in the non-DCI group. Since the non-DCI group is not considered a valid control group for HHH-therapy, no statistical analyses were performed on the differences in outcome parameters.

## Discussion

Poor-grade, unconscious SAH patients still suffer high risk of deterioration and development of DCI during the acute course after the initial bleed, despite early aneurysm repair and modern specialized NIC. The main therapeutic option remains the use of one or several components of HHH-therapy. The effectiveness of the HHH-therapy has been questioned, and according to recent literature, the limited scientific support favors only augmentation of systemic blood pressure [10]. Moreover, the use of excessive fluid overload and high doses of vasopressors in these patients carries the risk of cardiopulmonary and cerebral complications [21–23].

The use of HHH-therapy to resolve DCI, according to our standardized NIC protocol, is instituted cautiously to avoid negative side effects, and the treatment is frequently reevaluated in each patient. Systemic blood pressure targets are moderate to avoid excessive use of vasopressors. The establishment of bedside CBF XeCT in routine NIC provided an excellent possibility to evaluate the effects of HHH-therapy on CBF in our setting. A general problem in the scientific evaluation of HHH-therapy is the difficulty to obtain a relevant control group, since it is unethical to leave one group of patients with signs of DCI without treatment. To overcome this shortcoming to some extent, we identified non-DCI patients with XeCT measurements in the same time-windows to serve as a reference group, although CBF dynamics in these patients may differ from patients with suspicion of DCI.

At baseline, the subsequently HHH-treated DCI patients had lower CBF than non-DCI patients. During HHH treatment there was a significant elevation of global CBF, whereas CBF in non-DCI patients remained unchanged over time. There is a possibility that the observed increase during HHH-therapy may represent a natural spontaneous recovery of the initially low CBF, but there are several indications in this study that there was a real effect of the HHH-therapy on CBF, which is discussed in the following sections.

The most important therapeutic goal is to achieve better perfusion in near-ischemic regions, and therefore, analysis of regional blood flow is of special importance. There was an increase in regional CBF in the previously worst perfused vascular regions during HHH-therapy, and the proportion of ROI area with blood flow below the threshold of 20 ml/100 g/min decreased significantly. These findings indicate that the increase in CBF is not only luxury perfusion of already well-perfused regions, but also a true restoration of CBF in poorly perfused regions associated with HHH-therapy.

The restoration of CBF found in the group of patients receiving HHH-therapy does not necessarily correlate with better outcome. The absence of a strict DCI control group and the small number of patients make conclusions regarding outcome uncertain, and no long-term outcome data were analyzed. However, concerning short-term outcome, the frequencies of infarctions, and good clinical course outcome at discharge from the NIC unit were at similar levels for HHH-treated DCI patients and non-DCI patients. This observation also indicates that the HHH-therapy may have been effective.

One of the strengths of using XeCT with a mobile CT-scanner is the possibility to conduct CBF measurements bedside, which minimizes alterations in the physiological situation of the patients. The hemodynamic and respiratory parameters proved to be stable in this study, and the amount of sedation given was low (Table 2), which indicates that the results obtained were not confounded by those factors.

From this study, it is difficult to assess which component of the HHH-therapy was most important for the observed increase in CBF. However, the results indicate that the controlled hypervolemia and resulting reduction in hematocrit may be of importance, since the elevation of SBP was small and CPP not significantly increased. This indication is in contrast with previous studies suggesting that the hypertensive treatment is most important [10], which calls for further studies.

In evaluating the effect of HHH-therapy, baseline measurement of CBF should ideally be performed immediately prior to the initiation of the therapy. However, this was not possible for logistic reasons and the risk of delaying treatment. Baseline CBF in our study thus represents measurements performed at 0–48 h prior to the start of HHH-therapy, which is not optimal but reasonably close to the start of therapy. The second CBF measurement, during the five-day therapy, was performed at median 1.82 days (range 0.45–5.04 days) after the start of HHH-therapy, suggesting a sustained increase in CBF after initiation of the therapy. Early and repeated measurements would be preferred to better evaluate a direct relationship between HHH-therapy and CBF changes, but an increased number of measurements might be questionable due to radiation exposure and also logistically difficult.

We find bedside XeCT feasible for clinical use in NIC to estimate rCBF in unconscious, mechanically ventilated patients, but the procedure takes the resources of a trained team of intensive care and radiology personnel. Regarding safety issues, XeCT using 28% inhaled xenon is previously reported safe [24], and all patients in our study were physiologically stable during the procedures. Since inert and rapidly washed-out xenon is used as contrast agent, the method allows repeated measurements, although taking radiation exposure into consideration.

## Conclusions

The initial low global CBF found in patients diagnosed with DCI was significantly elevated during HHH-therapy. A concomitant increase in rCBF was also found in the worst perfused regions and the proportion of ROI area with blood flow below the threshold of 20 ml/100 g/min decreased significantly, which indicate a true effect of HHH-therapy. The increase in SBP was small, while the decrease in hematocrit was more pronounced, which may suggest that intravascular volume status and rheological effects are of importance. We found bedside XeCT to be a feasible and potentially clinically valuable tool in NIC.
